# Exploration of anti-inflammatory mechanism of forsythiaside A and forsythiaside B in CuSO_4_-induced inflammation in zebrafish by metabolomic and proteomic analyses

**DOI:** 10.1186/s12974-020-01855-9

**Published:** 2020-06-03

**Authors:** Lihong Gong, Linyuan Yu, Xiaohong Gong, Cheng Wang, Naihua Hu, Xuyang Dai, Cheng Peng, Yunxia Li

**Affiliations:** School of Pharmacy, Chengdu University of Traditional Chinese Medicine, Key Laboratory of Standardization for Chinese Herbal Medicine, Ministry of Education, National Key Laboratory Breeding Base of Systematic Research, Development and Utilization of Chinese Medicine Resources, Chengdu, 611137 China

**Keywords:** Forsythiaside A, Forsythiaside B, Zebrafish, Inflammation, Metabolomics, Proteomics

## Abstract

**Background:**

Inflammation is a general pathological phenomenon during severe disturbances to the homeostasis. Forsythiaside A (FA) and forsythiaside B (FB), isolated from the dried fruit of *Forsythia suspensa* (Thunb.) Vahl, are phenylethanoid compounds that show a significant anti-inflammatory effect. However, the properties and therapeutic mechanisms of this effect have not yet been systematically elucidated.

**Methods:**

In this study, the anti-inflammatory effects of FA and FB were investigated in CuSO_4_-induced inflammation in zebrafish larvae. Intracellular generation of reactive oxygen species (ROS) and nitric oxide (NO) was investigated using fluorescence probes. Metabolomic and proteomic analyses using liquid chromatography-mass spectrometry were carried out to identify the expressions of metabolites and proteins associated with the anti-inflammatory mechanism of FA and FB. Quantitative polymerase chain reaction (PCR) was performed to detect the progressive changes in gene expression.

**Results:**

FA and FB inhibited neutrophils migration to the damaged neuromasts and remarkably reduced CuSO_4_-induced ROS and NO generation in zebrafish larvae. Metabolomic analysis pointed to the involvement of nicotinate and nicotinamide metabolism, energy metabolism, pyrimidine metabolism, and purine metabolism. Proteomic analysis identified 146 differentially expressed proteins between the control and model groups. These included collagen [collagen type II alpha 1b precursor (col2a1b), collagen alpha-2(IX) chain precursor (col9a2), collagen type IX alpha I precursor (col9a1b)], nucleoside diphosphate kinase 3 isoform X1 (Nme3), WD repeat-containing protein 3 (Wdr3), and 28S ribosomal protein S7 mitochondrial precursor (Mrps7). FA and FB were shown to reverse the abnormal expressions of potential metabolite and protein biomarkers and alleviate CuSO_4_-induced damage to the neuromasts in the zebrafish lateral line.

**Conclusions:**

Our results indicate that FA and FB possess remarkable anti-inflammatory properties, protecting against CuSO_4_-induced neuromasts damage in zebrafish larvae. The results also suggest a multi-component and multi-regulatory therapeutic mechanism for FA and FB.

## Background

The inflammatory response is a key component in normal homeostasis that protects the body from irritation and restores damaged tissue structure and function. Generally, inflammatory reactions are beneficial to the body. However, excessive and uncontrolled inflammation can cause chronic diseases such as cancer and neurodegenerative diseases, including Parkinson's disease and Alzheimer's disease [1, 2]. During this process, over-activation of the macrophages and neutrophils can induce the secretion of interleukin-6 (IL-6), tumor necrosis factor-α (TNF-α), and interleukin-1β (IL-1β), which are significant mediators of the inflammatory response [3].

**Fig. 1:**
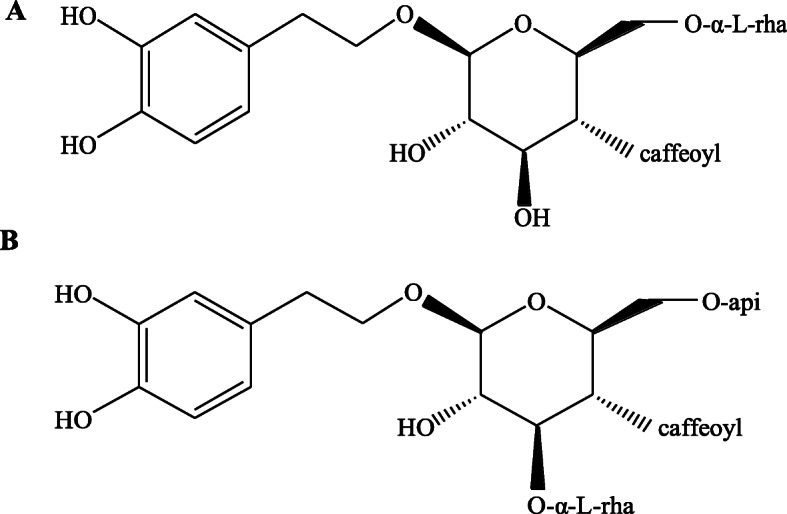
Chemical structures of forsythiaside A (**a**) and forsythiaside B (**b**)

Forsythiae Fructus is the dried fruit of *Forsythia suspensa* (Thunb.) Vahl. As an antipyretic and anti-inflammatory agent used in traditional Chinese medicine, Forsythiae Fructus has been used to treat various infectious diseases, such as acute nephritis and ulcers [4]. Forsythiaside A (FA, Fig. 1a) and forsythiaside B (FB, Fig. 1b) are the major bioactive components extracted from Forsythiae Fructus. They were reported to possess anti-inflammatory and anti-bacterial properties [5, 6]. However, few studies have been conducted to systematically investigate their anti-inflammatory properties and therapeutic mechanisms.

Zebrafish (*Danio rerio*), with morphological and physiological functions similar to humans, are being widely used in pharmacology research. It has unique advantages such as high fecundity, easy breeding, small size, availability of various transgenic lines, simple *in vivo* operation, and the possibility to perform real-time imaging [7–9]. The transparency of zebrafish larvae and the availability of transgenic zebrafish lines make it possible to monitor the inflammatory processes and observe cells behavior in vivo [10]. Studies have also shown that the zebrafish immune system shares significant similarities with humans, and almost all the human immune system cells have counterparts in zebrafish [11]. The neuromasts in the zebrafish lateral line system are composed of mechanosensory hair cells and can be damaged by physical or chemical stimulation [12]. CuSO_4_ is a metal chemical that induces neuromasts damage in zebrafish, followed by an infiltration of inflammatory cells, which in turn results in a progressive disruption of the neuromasts structure [13]. Unfortunately, the specific role of FA and FB in CuSO_4_-induced inflammation in zebrafish and the associated molecular mechanisms remain to be further explored.

The biological interpretation of common pharmacological research might be a great challenge due to the complicated biochemical regulatory mechanism at multiple levels. An integrated analysis of omics data is promising and can provide an improved comprehension of biological mechanisms by identifying the potential biomarkers and interpreting their intrinsic relations. The combination of metabolomics and proteomics is a powerful tool that frequently applied in complex mechanism exploration. Therefore, we investigated the anti-inflammatory mechanisms of FA and FB through an untargeted metabolic and proteomic profiling of zebrafish, using liquid chromatography-mass spectrometry. Based on the results, we propose a potential mechanism to explain the inflammatory behavior in zebrafish neuromasts during CuSO_4_ exposure, and systematically elucidate the anti-inflammatory effects and mechanisms of FA and FB. We thus expand the understanding of FA and FB in the treatment of inflammation and their potential clinical applications.

## Methods

### Materials

FA (MUST-18010303) and FB (MUST-18081202) were purchased from Chengdu MUST Bio-technology Co., Ltd. (Chengdu, China). Tricaine was obtained from Sigma-Aldrich (Shanghai, China). Acetonitrile, methanol, and formic acid of HPLC grade were collected from Merck Chemicals (Shanghai, China), Wokai Chemical Technology Co., Ltd. (Shanghai, China) and TCI Chemical Industry Development Co., Ltd. (Shanghai, China), respectively. DAF-FMDA and DCF-DA were purchased from Yeasen Bio-technology Co., Ltd. (Shanghai, China). TRIzol reagents were purchased from Ambion Life Technologies (Carlsbad, CA, USA). 5 × All-In-one MasterMix and Eva Green 2 × RT-qPCR MasterMix-Low RoX were purchased from Applied Biological Materials Inc. (Richomnd, BC, Canada). PCR primer sequences were synthesized in TSINGKE Biological Technology (Chengdu, China). Other chemicals and reagents used in this study were obtained from Kelong Chemical Reagent Factory (Chengdu, China).

### Zebrafish

Wild-type AB strain and neutrophil-specific transgenic (mpx: EGFP) zebrafish, in which neutrophils were labelled with an enhanced green fluorescent protein, were obtained from China Zebrafish Resource Center (Wuhan, China), and raised at 28.5 ± 1.0 °C on a 14-h light/10-h dark cycle. Zebrafish embryos were collected by natural spawning and maintained in the embryonic medium (5.00 mmol L^−1^ NaCl, 0.44 mmol L^−1^ CaCl_2_, 0.33 mmol L^−1^ MgSO_4_, 0.17 mmol L^−1^ KCl, and 0.1% methylene blue, equilibrated to pH 7.0) till drug administration [14, 15].

### Anti-inflammatory effects of FA and FB against CuSO_4_-induced inflammation in zebrafish

#### In vivo neutrophils recruitment assay

All experiments were carried out on 3 days post-fertilization (dpf) zebrafish larvae in the present study. Transgenic (mpx: EGFP) zebrafish larvae at 3 dpf were randomly transferred to a 12-well plate (15 larvae per well) and assigned to 8 groups for neutrophils recruitment investigation. In the control group, larvae were incubated in the embryonic medium. In the model group, larvae were exposed to 10 μmol L^−1^ CuSO_4_ for 40 min. In FA group, larvae were exposed to 1 h FA pre-treatment at 120, 60, 30 μmol L^−1^, followed by a mixture incubation of 10 μmol L^−1^ CuSO_4_ and FA (120, 60, 30 μmol L^−1^) for 40 min. In FB group, larvae were exposed to 1 h FB pre-treatment at 150, 75, 37.5 μmol L^−1^, followed by a mixture incubation of 10 μmol L^−1^ CuSO_4_ and FB (150, 75, 37.5 μmol L^−1^) for 40 min. All drugs were diluted in the embryonic medium. After the treatments, zebrafish larvae were washed with fresh medium and subsequently anesthetized with tricaine. Then, zebrafish larvae were photographed under a Leica M165Fic fluorescence microscope (Leica Microsystems, Germany). Finally, Image Pro Plus 6.0 software (Media Cybernetics, USA) was applied to quantify the recruitment of neutrophils to the zebrafish neuromasts.

#### Intracellular production of ROS and NO in zebrafish

2′,7′-dichlorodihydrofluorescein diacetate (DCF-DA) and diaminofluorophore 4-amino-5-methylamino-2′,7′-difluorofluorescein diacetate (DAF-FMDA) were used as fluorescence probes, respectively, to investigate intracellular reactive oxygen species (ROS) and nitric oxide (NO) accumulation in CuSO_4_-induced inflammation in zebrafish [16]. Wild-type AB strain zebrafish larvae were randomly transferred to a twelve-well plate (15 larvae per well), and assigned to eight groups following a previously-described method for modeling and drug administration. After the treatments, the zebrafish were moved to another twelve-well plate (15 larvae per well) and treated with the DCF-DA (0.05 μmol L^−1^) or DAF-FMDA (5 μmol L^−1^) solution. After 1 h incubation in the dark, the zebrafish larvae were washed with fresh medium and subsequently anesthetized with tricaine. The zebrafish larvae were photographed under a Leica M165Fic fluorescence microscope. Image Pro Plus 6.0 software was used to analyze the fluorescence intensity in individual zebrafish larvae to quantify the accumulation of ROS and NO.

#### Statistical analysis of neutrophils migration and ROS, NO accumulation

Zebrafish larvae were randomly assigned to each treatment group, and pharmacodynamic experiments were repeated three times. Statistical analyses were conducted by SPSS 25.0 (SPSS Inc., Chicago, IL, USA), and graphs were generated by GraphPad Prism 6.0 (GraphPad, San Diego, CA, USA). All statistics regarding neutrophils migration, ROS, and NO accumulation were evaluated by independent sample *t* test or Mann-Whitney *U* test (two groups) and one-way ANOVA or Kruskal-Wallis test followed by pairwise comparisons (three or more groups) depending on whether the data were normally distributed. Data were shown as mean ± SD and *p* < 0.05 were assumed for statistical significance indication.

### Metabolomic analysis

#### Sample processing and metabolites detection

Zebrafish larvae from the control, model, FA (120 μmol L^−1^), and FB (150 μmol L^−1^) groups were transferred to 2-mL centrifuge tubes, respectively. We then added 1 mL 80% methanol and steel balls to the tubes. Samples were ground for 1 min in a Tissue Grinding Device (SCIENTZ-48, Xinzhi Biotechnology Co., Ltd.) at 70 Hz. Samples were then placed in an Ultrasonic Machine (KW-100TDV, Shumei Ultrasonic Instrument Co., Ltd.) at room temperature for 30 min and subsequently incubated on ice for 30 min. After being centrifuged for 10 min (14,000 rpm, 4 °C), the supernatant was transferred to a new tube. Samples were dried by vacuum concentration and then dissolved with 400 μL 2-chlorobenzalanine methanol aqueous solution (1:1, 4 °C). Samples were filtered through a 0.22-μm microfilter membrane and then prepared for liquid chromatography-mass spectrometry (LC-MS) detection. Quality control samples were obtained by mixing a 20-μL aliquot from each sample together.

Chromatographic separation was performed on a High-performance liquid chromatography (HPLC, Ultimate 3000 system, Thermo), equipped with a Waters column (1.8 μm, 150 × 2.1 mm) at 40 °C. The autosampler temperature was set at 8 °C. Gradient elution of analytes was carried out at a flow rate of 0.25 mL min^−1^ with 5 mmol L^−1^ ammonium formate in water (A) and acetonitrile (B) in the negative model or with 0.1% formic acid in water (C) and 0.1% formic acid in acetonitrile (D) in the positive model. Two microliter samples were injected after equilibration. A linear gradient of solvent B (negative model) or solvent D (positive model) was programmed as follows: 0–1 min, 2% B or 0.1% D; 1–9 min, 2–50% B/D; 9–12 min, 50–98% B/D; 12–13.5 min, 98% B/D; 13.5–14 min, 98–2% B/D; 14–20 min, 2% D or 14–17 min, 2% B.

The electrospray ionization mass detection (ESI-MS^n^) in positive and negative ion modes was performed on a Mass spectrometer (Q Exactive Focus, Thermo) with a spray voltage of 3.8 kV and − 2.5 kV, respectively. Sheath gas and auxiliary gas were set at 45 and 15 arbitrary units, respectively. The capillary temperature was set at 325 °C . The Orbitrap Analyzer (Orbitrap Fusion Lumos mass spectrometer, CA, USA) performed a full scan with a mass to charge ratio (m/z) range of 81–1000 and a mass resolution of 70,000. Data-dependent experiments were performed on mass spectrometry/mass spectrometry (MS/MS) with higher-energy collisional dissociation (HCD) scanning mode. The standard collision energy was 30 eV. Dynamic exclusion was implemented to remove some unnecessary information from the MS/MS spectrum.

#### Data processing and multivariate analyses of metabolites

Data from the ultra-performance liquid chromatography-mass spectrometry/mass spectrometry (UPLC-MS/MS) were displayed in mzXML format using the ProteoWizard software (v3.0.8789). Data pre-processing was performed by the XCMS package of R language (v3.3.2) using autoscaling, mean-centering, and scaling to unit variance. Parameters were set as follows: bw = 2, ppm = 15, mzwid = 0.015, peak width = c (5, 30), mzdiff = 0.01, method = cent wave. The area of metabolite peaks in each sample was normalized by the summation method applied in Metaboanalyst (http://www.metaboanalyst.ca/). Multivariate analyses of metabolites, including standard peak areas and retention time, were performed using the R language to further locate the specific metabolites in the dataset. A biomarker was generated and queried for accurate molecular weights, with mass errors of less than 30 part per million (ppm). The fracture patterns of potential biomarkers were analyzed, and their identification was performed using the Human Metabolome Database (HMDB) (http://www.hmdb.ca) and METLIN (http://metlin.scripps.edu/). The Kyoto Encyclopedia of Genes and Genomes Database (KEGG) (http://www.kegg.jp/) was used to plot a metabolomic pathway network diagram. Using Metaboanalyst, we performed a pathway enrichment analysis to screen metabolomic pathways of the identified metabolites in the current study.

#### Statistical analysis of metabolomics

In metabolomic analysis, 10 biological replications were made in each group in the present study. Orthogonal projections to latent structures discriminant analysis (OPLS-DA) was used to filter metabolites, and variable importance in the projection (VIP) values were used to promote group discrimination. The S-Plot (R language: v3.3.2) was applied to detect the differential metabolites with values of VIP ≥ 1. One-way ANOVA of SPSS 25.0 was conducted to determine whether the potential metabolites were significantly different (*p* ≤ 0.05) among the control, model, FA, and FB groups. Finally, the metabolites with VIP ≥ 1 and *p* ≤ 0.05 were considered as potential metabolite biomarkers [17, 18].

### Proteomic analysis

#### Protein extraction and digestion

Proteins were extracted from zebrafish larvae of the control, model, FA (120 μmol L^−1^), and FB (150 μmol L^−1^) groups, as previously described [19]. Briefly, 500 μL lysis buffer (2% sodium deoxycholate, 50 mM ammonium bicarbonate, 75 mM sodium chloride) was added to the samples, which were then ground on ice in an Ultrasonic Crushing Machine (Scientz-JY92, Ningbo Xinzhi Biotechnology Co., Ltd.) for 10 min, with cycles of 2 s on and 4 s off, at 15% power. The ground material was centrifuged for 10 min (10,000×*g*, 4 °C). We then added 10 mM dithiothreitol to the supernatant to precipitate the proteins at − 20 °C . The precipitation procedure was repeated with acetone until the supernatant became colorless, as previously described [20]. Then, the supernatant was incubated at 56 °C for 1 h and subsequently alkylated with 55 mmol L^−1^ iodoacetamide for 45 min in the dark at room temperature. Proteins were then resuspended in the lysis buffer, and the concentration of protein was detected using the BCA assay. The protein solution (100 μg) was digested by Trypsin Gold (40:1, protein:trypsin) at 37 °C overnight. Peptides desalination was carried out using a Strata X C_18_ column, and the samples were then vacuum-dried following the manufacturer's instructions.

#### TMT labeling and fractionation

The peptides were labeled using Tandem Mass Tags (TMT) six plex Isobaric Label Reagent Set (Thermo Scientific, 90061) following the manufacturer’s instructions. Samples of the control and model groups were labeled with tags 126 and 127, respectively, while samples of FA and FB groups were labeled with tags 128 and 129, respectively. The labeled peptides were separated using a Shimadzu LC-20AB HPLC Pump system (Shimadzu, Kyoto, Japan), equipped with a high-pH reversed-phase (RP) column (5 μm, Phenomenex, CA, USA). The labeled peptides were firstly reconstituted to 2 mL with buffer A (5% ACN and 95% H_2_O, pH adjusted to 9.8 with ammonia) and then loaded on the column for separation with a gradient of 5% buffer B (5% H_2_O and 95% ACN, pH adjusted to 9.8 with ammonia) for 10 min, 5–35% buffer B for 40 min, and 35–95% buffer B for 1 min at a flow rate of 1 mL min^−1^. Finally, the system was maintained for 3 min at this condition, followed by a decrease to 5% buffer B within 1 min and equilibration in 5% buffer B for 10 min. The elution process was monitored by measuring absorbance at 214 nm. Fractions were collected every minute and pooled as 20 fractions. The eluted peptides were subsequently concentrated by vacuum centrifugation.

#### LC-MS/MS analysis

The fractions were resuspended in buffer A (2% acetonitrile and 0.1% formic acid) and then centrifuged for 10 min (20,000×*g*). The supernatants were loaded onto a C_18_ trap column on an LC-20 AD nano-HPLC instrument. An internally packed analytical C_18_ column was used to elute and separate the peptides. The elution was performed at a flow rate of 600 nL min^−1^ under the following conditions: 8–35% buffer B (5% H_2_O and 95% ACN, pH adjusted to 9.8 with ammonia), 0–35.00 min; 60% buffer B, 35.01–40.00 min; 80% buffer B, 40.01–45.00 min; 5% buffer B, 45.01–45.10 min. The equilibration time was 10 min. The eluted peptides were subjected to nano-electrospray ionization, followed by MS/MS (Orbitrap Fusion Lumos mass spectrometer, CA, USA) and nano HPLC analyses. The mass spectrometry analyses were performed with a scan range of 350–1800 m/z, and the survey scans were obtained at 120,000 mass resolution of 400 m/z using an Orbitrap analyzer. Dynamic exclusion parameters were a repeat count of 2 and a repeat duration of 30 s.

#### Data processing and proteins identification

The Proteome Discoverer software v2.1 (Thermo Fisher Scientific, MA, USA) was applied to process and quantify the raw data files. A protein search was performed in the RefSeq human protein database (24078 sequences, released in 2017) based on the SEQUEST algorithm. Parameters were set as follows: fixed modifications, including carbamidomethylation of cysteine, and modification at N-terminus and K of TMT six-plex. Methionine oxidation was applied as variable modification. Trypsin was set as the enzyme, allowing two missing cleavages. Verification and identification were performed using the Percolator software (Proteome Discoverer sequest (v2.1)). The protein and peptide profile matching the false discovery rate (FDR) was set at 0.01 [21]. Total protein intensity was generated by summing all reported ion intensities of unique peptides that matched each protein. Corrections of the bias and background were performed by checking protein quantification and normalization. Proteins containing at least two unique peptides were further quantified.

#### Statistical analysis of proteomics

In proteomic analysis, proteins were extracted from three biological replications in each group. The fold changes of proteins in the control, model, FA, and FB groups were calculated as mean value according to the relative and absolute quantification ratio of the protein isobaric tags. Student’s *t* tests of SPSS 25.0 were performed to further determine whether the differential proteins were significantly different (*p* < 0.05) between groups. Accordingly, proteins with *p* < 0.05 and fold change > 1.2 were identified as differentially expressed biomarkers.

### Interactive network construction of metabolites and proteins

For pathway and network analyses, the correlation coefficient and *p* value of differentially expressed metabolites and proteins (with NCBI IDs) were calculated by the R language. The metabolites and proteins with *p* < 0.05 and absolute correlation coefficient > 0.9 were screened out, and further imported into Cytoscape software (v3.4.0) to generate the final association analysis network diagram.

### Reverse transcription quantitative real-time PCR (RT-qPCR) analysis

We used RT-qPCR to detect and quantify the mRNA expressions of nucleoside diphosphate kinase 3 isoform X1 (Nme3), WD repeat-containing protein 3 (Wdr3), 28S ribosomal protein S7 mitochondrial precursor (Mrps7), and Collagen. We also quantified the inflammatory mediators such as IL-6, IL-1β, TNF-α, and genes involved in nuclear transcription factor-kappa B (NF-κB), mitogen-activated protein kinases (MAPK), and Janus kinase/signal transducer and activator of transcription (JAK-STAT) signaling pathways. In brief, zebrafish larvae were washed three times with RNase-free water and homogenized with the TRIzol reagent. Homogenized tissues were extracted by chloroform and centrifuged for 15 min (12,000×*g*, 4 °C). The supernatant was transferred to a new tube, and isopropyl alcohol of equal volume was added for RNA precipitation. After being centrifuged for 10 min (12,000×*g*, 4 °C), 75% ethanol was used to wash the pellet, which was then dried and then suspended in 50 μL RNase-free water. The optical density (OD) at 260/280 nm was measured for RNA purity detection. The RT-qPCR reaction conditions were set as follows: 95 °C for 10 min, 40 cycles of 95 °C for 15 s and 60 °C for 30 s. The relative mRNA expressions levels were calculated by the 2^−ΔΔCT^ method. All primers used for gene amplification were designed using the Primer-BLAST (NCBI), and their sequences were listed in Table S1. For gene expression analysis, one-way ANOVA of SPSS 25.0 was used for statistical comparisons. Data were shown as mean ± S.D and *p* < 0.05 were assumed for the probability level for statistical significance.

## Results

### Inhibitory effects of FA and FB against CuSO_4_-induced inflammation in zebrafish

**Fig. 2:**
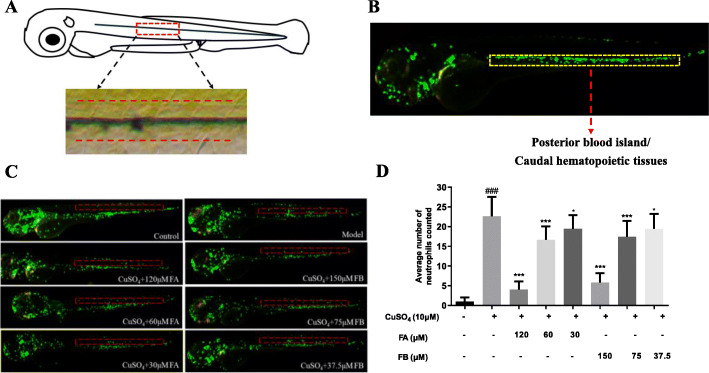
FA and FB inhibited the migration of neutrophils to the injury site in CuSO_4_-treated zebrafish. **a** A general view of the zebrafish larvae at 3 dpf. The boxed area represented the horizontal lateral line, and neutrophils within the horizontal line (dotted red lines) were calculated in quantification experiments. **b** The area of the posterior blood island or caudal hematopoietic tissues (the boxed region with dotted yellow lines) in an untreated transgenic (mpx: EGFP) zebrafish. Most leukocytes in this development stage were distributed in this area. **c** Microphotographs exhibited neutrophils migration in the control, model, FA, and FB groups (neutrophils of 3 dpf transgenic (mpx: EGFP) zebrafish exhibiting green fluorescence). The red dotted lines represented the location of the horizontal muscles of zebrafish. **d** FA and FB dose-dependently reduced the number of neutrophils recruited in the injury site. ^#^*p* < 0.05, ^##^*p* < 0.01, ^###^*p* < 0.001, compared with the control group; ^*^p < 0.05, ^**^p < 0.01, ^***^p < 0.001, compared with the model group. Data were shown as mean ± S.D, *n* = 35

The primary lateral line system of zebrafish was established within 3 days after fertilization. Studies have previously reported that the addition of CuSO_4_ can swiftly destroy the hair cells in zebrafish lateral line neuromasts through oxidation and cell death [22]. The zebrafish larvae of each treatment group were observed using a fluorescence microscope, and the number of fluorescent cells in the region of about ten cell diameters within the horizontal muscle was counted (Fig. 2a). In the control group, most of the neutrophils were assembled in the posterior blood island or caudal hematopoietic tissue, in which most leukocytes were distributed in this development stage (Fig. 2b). In contrast, zebrafish larvae exposed to CuSO_4_ for 40 min formed a typical neutrophils cluster in the horizontal muscles, which indicated that neutrophils could migrate to the lateral line neuromasts, with the induction of CuSO_4_. On the contrary, co-treatment with FA or FB inhibited neutrophils migration and decreased the assembled number of neutrophils, as shown in Fig. 2c (the red dotted lines represented the location of the horizontal muscles). These results indicated that FA and FB could dose-dependently inhibit the migration of neutrophils to the zebrafish lateral line neuromasts (Fig. 2d).

### Inhibitory effects of FA and FB against CuSO_4_-induced intracellular ROS and NO generation in zebrafish

**Fig. 3:**
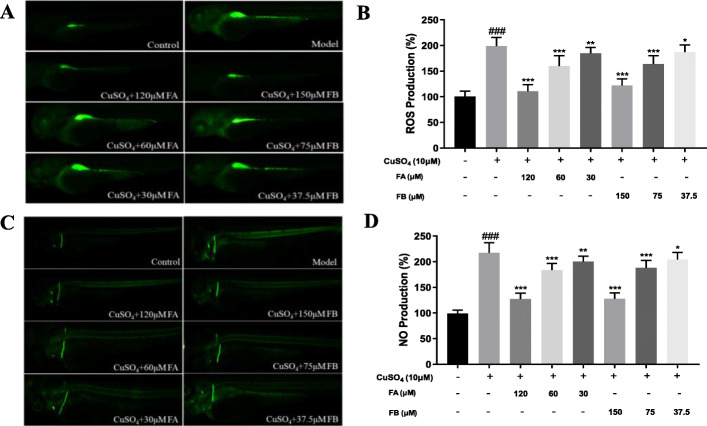
FA and FB alleviated inflammation in zebrafish by inhibiting CuSO_4_-induced ROS and NO production. **a** Microphotographs exhibited ROS production in the control, model, FA, and FB groups (3 dpf wild-type AB strain zebrafish larvae). **b** FA and FB dose-dependently reduced ROS generation induced by CuSO_4_. **c** Microphotographs exhibited the production of NO in the control, model, FA, and FB groups (3 dpf wild-type AB strain zebrafish larvae). **d** FA and FB dose-dependently reduced NO generation induced by CuSO_4_. ^#^*p* < 0.05, ^##^*p* < 0.01, ^###^*p* < 0.001, compared with the control group; ^*^p < 0.05, ^**^p < 0.01, ^***^p < 0.001, compared with the model group. Data were shown as mean ± S.D, *n* = 35

The overproduction of ROS by polymorphonuclear neutrophils at inflammatory sites might cause endothelial dysfunction and tissue damage [23]. Therefore, DCF-DA was used to detect ROS production in CuSO_4_-induced inflammation in zebrafish. Fig. 3a was a representative image of intracellular ROS production. The control group, not treated with CuSO_4_, FA, or FB, exhibited a dark and weak fluorescent image. However, the model group exposed to CuSO_4_ presented a brighter and stronger fluorescent image. Thus, the addition of CuSO_4_ could lead to intracellular ROS generation in zebrafish. However, FA and FB dose-dependently decreased intracellular ROS accumulation in zebrafish (Fig. 3b).

Besides, NO is also a common conduction molecular and plays an essential role in both the initiation and development of inflammation [24]. Therefore, the fluorescence probe DAF-FMDA was performed to measure the intracellular NO production induced by CuSO_4_, for further evaluating the inhibitory effects of FA and FB in zebrafish. As a result, zebrafish larvae exposed to CuSO_4_ exhibited a high generation of NO (Fig. 3c). On the contrary, zebrafish larvae treated with FA or FB showed a significant decrease of NO accumulation in a dose-dependent manner (Fig. 3d). These results indicated that FA and FB could relieve CuSO_4_-induced inflammation in zebrafish by inhibiting the intracellular generations of ROS and NO.

### Metabolomics

#### Multivariate analyses of UPLC-MS/MS data

**Fig. 4:**
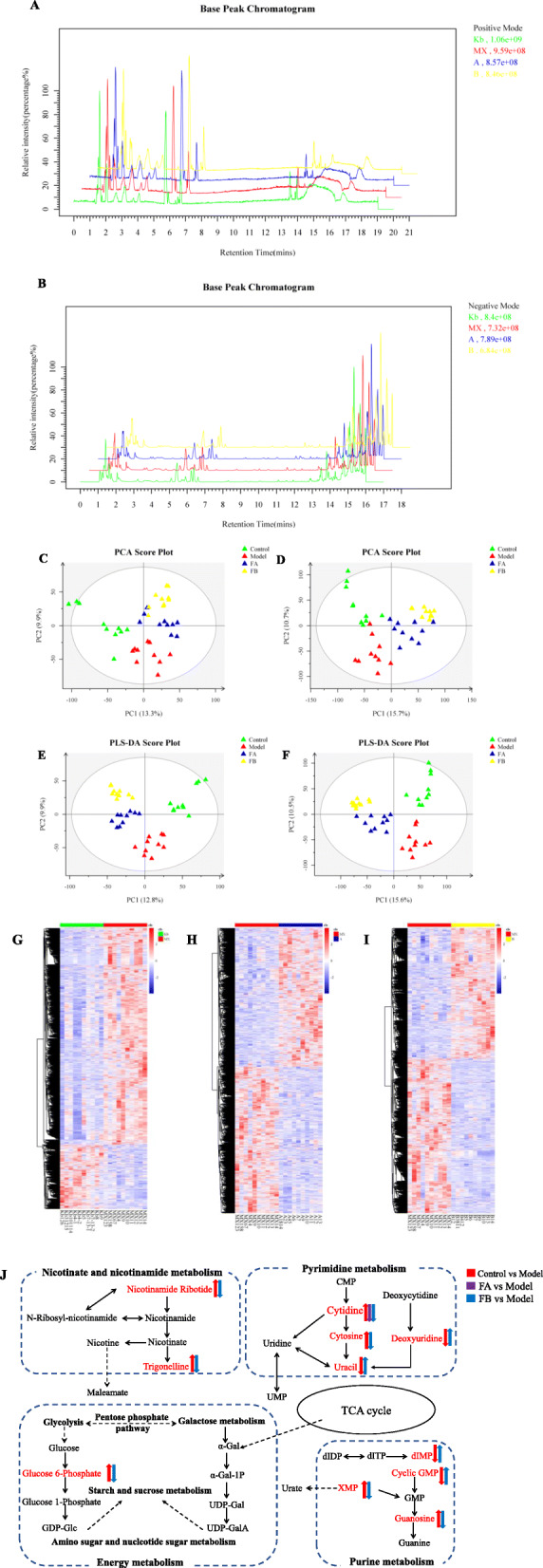
Metabolomic analyses of zebrafish larvae samples from the control, model, FA, and FB groups. Representative base peak chromatogram (BPC) of the control, model, FA, and FB groups in the positive ion mode (**a**) and negative ion mode (**b**). PCA score plot of metabolites in the positive ion mode (**c**) and negative ion mode (**d**). PLS-DA score plot of metabolites in the positive ion mode (**e**) and negative ion mode (**f**). Heat-map of differential metabolites of control vs. model (**g**), model vs. FA (**h**), and model vs. FB (**i**). Rows: differential metabolites; Columns: zebrafish larvae samples. The rectangle in different colors represented the expression level of metabolites: the highest (red), the lowest (blue), and the mean (white). **j** Metabolic pathways (bold) participating in the anti-inflammatory process of FA and FB against CuSO_4_-induced inflammation in zebrafish. The metabolites (red) were the identified biomarkers in the present study. Arrows near metabolites indicated the biomarkers’ relative expressions of control vs. model, model vs. FA, and model vs. FB

UPLC-MS/MS was used to analyze all zebrafish larvae samples in both positive and negative ion modes. Representative base peak chromatograms (BPC) of zebrafish larvae samples from the control, model, FA, and FB groups were obtained under optimal conditions (Fig. 4a, b). Fifteen thousand four hundred eighty-nine variables (ESI^+^) and 26308 variables (ESI^−^) could simultaneously be detected in 20 min.

Multivariate analyses suggested clear separations from the control, model, FA, and FB groups. The principal component analysis (PCA) model and the profiles of the control, model, FA, and FB groups showed a tendency towards separation (Fig. 4c, d). The partial least squares-discriminant analysis (PLS-DA) was further performed to distinguish the differences of metabolites in the four groups, and the scores plot of PLS-DA indicated that these groups could be obviously distinguished (Fig. 4e, f). It was found that the control and model groups were separated in both positive mode and negative mode, indicating that a CuSO_4_-induced acute inflammation model in zebrafish larvae was successfully established. FA (120 μmol L^−1^) and FB (150 μmol L^−1^) groups were separated from the model group and were closer to the control group, which highlighted the anti-inflammatory effects of drugs.

#### Identification and analysis of metabolites

The heat-maps of metabolites changes of control vs. model, model vs. FA, and model vs. FB were shown in Fig. 4g–i. Based on the outcome of MS/MS and the information from online database, we carried out the identification of these metabolites. As a result, a total of 88 metabolites were found to be significantly altered in the model group relative to the control group. Treatment with FA and FB reversed the changes of 14 and 35 metabolic biomarkers, respectively. As listed in Table 1, 36 metabolic biomarkers were found to be involved in CuSO_4_-induced inflammation after treatment with FA or FB. Both FA and FB could reverse the abnormal expressions of the following metabolites involved in inflammatory response: uridine 5-diphosphate, carnitine, Val-Abu-OH, ciliatine, cytidine, benzaldehyde, isobutyryl carnitine, N-acetyl-L-phenylalanine, gamma-glutamyl-leucine, dihydrojasmonic acid, cyclic GMP, 12-hydroxydodecanoic acid, and N-arachidonoyl dihydroxypropylamine. FA and FB might exert anti-inflammatory effects by regulating the expressions of these metabolites [25–30].

**Table 1:** Identification results and change trends of important differential metabolites

No.	Metabolities	m/z	Rt/s	ESI mode	Formula	VIP	*p* value	Control vs model	Model vs FA	Model vs FB
1	Uridine 5-diphosphate	402.99	71.91	−	C_9_H_14_N_2_O_12_P_2_	1.793	0.001	↑^###^	↓^**^	↓^***^
2	XMP	363.03	75.89	−	C_10_H_13_N_4_O_9_P	2.133	0.000	↑^###^	─	↓^***^
3	Pipecolic acid	130.08	78.20	+	C_6_H_11_NO_2_	1.242	0.034	↑^#^	─	↓^*^
4	Carnitine	162.11	91.92	+	C_7_H_16_NO_3_	1.288	0.027	↑^#^	↓^*^	↓^*^
5	5-methoxyindoleacetate	206.08	97.77	+	C_11_H_11_NO_3_	1.728	0.001	↑^##^	─	↓^*^
6	Cytosine	112.05	101.80	+	C_4_H_5_N_3_O	1.295	0.026	↑^#^	─	↓^*^
7	Nicotinamide ribotide	335.06	102.13	+	C_11_H_15_N_2_O_8_P	1.990	0.000	↑^###^	─	↓^***^
8	Trigonelline	138.05	102.30	+	C_7_H_7_NO_2_	1.592	0.004	↑^##^	─	↓^*^
9	1-aminocyclohexanecarboxylic acid	144.10	102.86	+	C_7_H_13_NO_2_	1.787	0.001	↑^###^	─	↓^*^
10	N-alpha-acetyllysine	189.12	104.90	+	C_8_H_16_N_2_O_3_	1.275	0.028	↑^#^	─	↓^*^
11	Val-Abu-OH	309.11	105.87	−	C_14_H_18_N_2_O_6_	1.655	0.003	↑^##^	↓^*^	↓^**^
12	Chitobiose	425.17	107.48	+	C_16_H_28_N_2_O_11_	1.940	0.000	↑^###^	↓^**^	─
13	Glutamylthreonine	249.11	110.44	+	C_9_H_16_N_2_O_6_	1.477	0.009	↑^##^	─	↓^***^
14	Glucose 6-phosphate	261.03	112.61	+	C_6_H_13_O_9_P	1.458	0.000	↑^#^	─	↓^***^
15	dIMP	331.04	115.74	−	C_10_H_13_N_4_O_7_P	1.280	0.029	↓^#^	─	↑^**^
16	Ciliatine	126.03	118.96	+	C_2_H_8_NO_3_P	1.180	0.000	↑^###^	↓^***^	↓^***^
17	N6-acetyl-L-lysine	189.12	121.39	+	C_8_H_16_N_2_O_3_	1.193	0.042	↑^#^	─	↓^*^
18	N-A-acetyl-L-arginine	217.13	121.98	+	C_8_H_16_N_4_O_3_	1.751	0.001	↑^##^	─	↓^**^
19	Uracil	113.03	139.01	+	C_4_H_4_N_2_O_2_	1.645	0.003	↓^##^	─	↑^*^
20	Cytidine	242.08	174.43	−	C_9_H_13_N_3_O_5_	1.464	0.011	↑^#^	↓^*^	↓^**^
21	Guanosine	282.08	230.23	−	C_10_H_13_N_5_O_5_	1.399	0.015	↑^#^	─	↓^***^
22	Deoxyuridine	227.06	289.06	−	C_9_H_12_N_2_O_5_	1.363	0.019	↓^#^	─	↑^*^
23	L-beta-homomethionine	164.07	336.67	+	C_6_H_13_NO_2_S	1.215	0.038	↑^#^	─	↓^**^
24	Gamma-glutamyl-methionine	279.10	337.22	+	C_10_H_19_N_3_O_4_S	2.147	0.000	↑^###^	─	↓^***^
25	Benzaldehyde	107.05	343.55	+	C_7_H_6_O	1.794	0.001	↑^##^	↓^**^	↓^**^
26	Isobutyryl carnitine	232.15	373.56	+	C_11_H_21_NO_4_	1.208	0.040	↑^#^	↓^*^	↓^*^
27	N-acetyl-L-Phenylalanine	206.08	385.01	−	C_11_H_13_NO_3_	1.590	0.005	↑^##^	↓^*^	↓^**^
28	Gamma-glutamyl-leucine	261.14	404.26	+	C_11_H_21_N_3_O_4_	1.904	0.000	↑^###^	↓^**^	↓^**^
29	Jasmonate	209.12	521.42	−	C_12_H_18_O_3_	1.045	0.000	↓^##^	─	↑^***^
30	N-acetyltryptophan	247.11	543.58	+	C_13_H_14_N_2_O_3_	1.478	0.010	↑^##^	─	↓^**^
31	Dihydrojasmonic acid	211.13	601.25	−	C_12_H_20_O_3_	1.348	0.020	↓^#^	↑^*^	↑^**^
32	Cyclic GMP	344.07	625.18	−	C_10_H_12_N_5_O_7_P	1.731	0.002	↓^##^	↑^***^	↑^**^
33	12-hydroxydodecanoic acid	215.16	710.07	−	C_12_H_24_O_3_	1.780	0.001	↑^##^	↓^*^	↓^*^
34	NAGly	360.25	794.64	−	C_22_H_35_NO_3_	2.005	0.000	↑^###^	─	↓^**^
35	L-A-lysophosphatidylserine	524.30	823.98	−	C_24_H_48_NO_9_P	1.246	0.034	↓^#^	─	↑^**^
36	N-arachidonoyl dihydroxypropylamine	376.29	825.78	−	C_23_H_39_NO_3_	1.643	0.003	↓^##^	↑^**^	↑^**^

↑, the metabolite was up-regulated; ↓, the metabolite was down-regulated

^#^*p* < 0.05, ^##^*p* < 0.01, ^###^*p* < 0.001, compared with the control group

^*^p < 0.05, ^**^p < 0.01, ^***^p < 0.001, compared with the model group

Based on the information of the KEGG database, we performed the topology and pathway enrichment analyses to identify the involved metabolomic pathways. We also applied the metaboanalyst to calculate the −log(p) value and the pathway impact value based on pathway enrichment and topology analyses, respectively. According to the values of −log(p) and pathway impact, we finally identified the potential pathways associated with the effect of FA and FB in CuSO_4_-induced inflammation, summarized in Table S2. The identified biomarkers and related pathways were shown in Fig. 4j. Our findings pointed mainly to the involvement of nicotinate and nicotinamide metabolism, energy metabolism, pyrimidine metabolism, and purine metabolism.

### Proteomics

#### Proteins identification

UPLC-MS/MS was applied to analyze the proteomic profiles of CuSO_4_-induced inflammation in zebrafish after FA and FB treatment. As a result, 5212 proteins were detected, and 2976 proteins were further identified and quantified with at least two unique peptides and the false discovery rate (FDR) < 1%. According to the criteria of fold change > 1.2 and *p* < 0.05, there were 146 differentially expressed proteins (DEPs) in the model group, including 60 upregulated proteins and 86 downregulated proteins, compared with the control group. Additionally, FA and FB treatment reversed the expressions of 51 and 18 DEPs, respectively (Table 2).

**Table 2:** Dysregulated proteins in different groups

No.	Accession	Gene Symbol	Description	Control vs model	Model vs FA	Model vs FB
1	NP_001014348	srpx	Sushi repeat-containing protein SRPX precursor	↓^##^	↑^**^	↑^*^
2	NP_001028768	mrps7	28S ribosomal protein S7, mitochondrial precursor	↑^###^	↓^***^	↓^***^
3	NP_001077285	zgc:162509	Uncharacterized protein LOC553299	↑^##^	↓^**^	↓^**^
4	NP_001268407	col2a1b	Collagen, type II, alpha 1b precursor	↑^##^	↓^**^	↓^***^
5	NP_571742	gch2	GTP cyclohydrolase 1	↑^##^	↓^***^	↓^**^
6	NP_942574	wdr3	WD repeat-containing protein 3	↑^##^	↓^*^	↓^*^
7	NP_956140	ptpn11a	Tyrosine-protein phosphatase non-receptor type 11	↑^#^	↓^*^	↓^*^
8	NP_956258	rcvrn2	Recoverin 2	↓^#^	↑^**^	↑^**^
9	NP_991174	nit2	Omega-amidase NIT2	↑^#^	↓^*^	↓^*^
10	NP_997744	col9a2	Collagen alpha-2(IX) chain precursor	↓^##^	↑^***^	↑^***^
11	NP_998429	col9a1b	Collagen type IX alpha I precursor	↑^##^	↓^**^	↓^***^
12	XP_021324964	nme3	Nucleoside diphosphate kinase 3 isoform X1	↑^###^	↓^***^	↓^***^
13	XP_695887	scaf4a	Splicing factor, arginine/serine-rich 15	↓^#^	↑^*^	↑^**^
14	NP_001002461	txn	Thioredoxin	↓^##^	↑^*^	─
15	NP_001003625	nup85	Nuclear pore complex protein Nup85	↑^#^	↓^**^	─
16	NP_001004660	snrpg	Small nuclear ribonucleoprotein G isoform 2	↓^#^	↑^*^	─
17	NP_001006043	ctsz	Cathepsin Z precursor	↓^#^	↑^*^	─
18	NP_001017899	capns1a	Calpain small subunit 1	↓^#^	↑^**^	─
19	NP_001019906	eif3m	Eukaryotic translation initiation factor 3 subunit M	↓^##^	↑^**^	─
20	NP_001020680	ppp6r2a	Serine/threonine-protein phosphatase 6 regulatory subunit 2	↑^##^	↓^**^	─
21	NP_001038800	rpl22l1	60S ribosomal protein L22-like 1	↓^###^	↑^***^	─
22	NP_001071203	bop1	Ribosome biogenesis protein bop1	↑^#^	↓^**^	─
23	NP_001082840	mrps30	39S ribosomal protein S30, mitochondrial	↓^#^	↑^*^	─
24	NP_001093210	matn1	Cartilage matrix protein precursor	↓^#^	↑^***^	─
25	NP_001096604	crygm2d16	Crystallin, gamma M2d16	↓^##^	↑^***^	─
26	NP_001103591	rps23	40S ribosomal protein S23	↓^#^	↑^*^	─
27	NP_001166027	bxdc2	Ribosome biogenesis protein BRX1 homolog	↑^##^	↓^**^	─
28	NP_001289671	atp5f1e	ATP synthase subunit epsilon, mitochondrial	↓^##^	↑^**^	─
29	NP_001338629	LOC100330864	Ribonucleoside-diphosphate reductase subunit M2 isoform 1	↑^#^	↓^*^	─
30	NP_571328	opn1mw1	Green-sensitive opsin-1	↓^##^	↑^*^	─
31	NP_956159	cdc42l	Cell division control protein 42 homolog	↓^#^	↑^*^	─
32	NP_956500	erap1b	Endoplasmic reticulum aminopeptidase 1 precursor	↓^#^	↑^*^	─
33	NP_957036	rps26l	Ribosomal protein S26	↓^#^	↑^**^	─
34	NP_957153	slc25a20	Mitochondrial carnitine/acylcarnitine carrier protein	↓^#^	↑^**^	─
35	NP_958493	pfn2	Profilin 2	↓^##^	↑^*^	─
36	NP_958500	arpc1a	Actin-related protein 2/3 complex subunit 1A	↓^#^	↑^**^	─
37	NP_963878	rpl12	60S ribosomal protein L12	↓^##^	↑^*^	─
38	NP_991317	psmd10	26S proteasome non-ATPase regulatory subunit 10	↓^#^	↑^*^	─
39	NP_997743	mcmbp	Mini-chromosome maintenance complex-binding protein	↓^##^	↑^*^	─
40	NP_997750	ist1	IST1 homolog isoform 1	↓^##^	↑^***^	─
41	NP_998569	lipf	Lysosomal acid lipase/cholesteryl ester hydrolase precursor	↓^#^	↑^*^	─
42	NP_998605	plrg1	Pleiotropic regulator 1	↑^##^	↓^**^	─
43	NP_999858	lgals3b	Galectin-3	↓^##^	↑^***^	─
44	NP_999977	epb41l3b	Erythrocyte membrane protein band 4.1-like 3b	↑^#^	↓^*^	─
45	XP_001923961	nxf1	Nuclear RNA export factor 1	↑^##^	↓^*^	─
46	XP_005159500	col11a2	Collagen alpha-2(XI) chain isoform X1	↓^##^	↑^*^	─
47	XP_005163206	tnni2a.4	Troponin I, skeletal, fast 2a.4 isoform X1	↑^##^	↓^***^	─
48	XP_017208249	coro6	Coronin-6 isoform X1	↓^#^	↑^***^	─
49	XP_021322373	arhgef1b	Rho guanine nucleotide exchange factor 1 isoform X1	↓^##^	↑^**^	─
50	XP_685270	sgca	Alpha-sarcoglycan	↑^#^	↓^**^	─
51	XP_691943	si:dkey-23a13.8	Histone H2B 1/2-like	↑^##^	↓^**^	─
52	NP_001019612	psma2	Proteasome alpha 2 subunit	↑^#^	─	↓^*^
53	NP_001071216	scg2b	Secretogranin-2 precursor	↓^##^	─	↑^**^
54	NP_001313480	si:dkey-251i10.2	si:dkey-251i10.2 precursor	↓^#^	─	↑^*^
55	NP_957419	spag7	Sperm-associated antigen 7 homolog	↑^#^	─	↓^*^
56	XP_005155974	si:ch211-222l21.1	Prothymosin alpha	↑^###^	─	↓^**^

↑, the protein was up-regulated; ↓, the protein was down-regulated

^#^*p* < 0.05, ^##^*p* < 0.01, ^###^*p* < 0.001, compared with the control group

^*^p < 0.05, ^**^p < 0.01, ^***^p < 0.001, compared with the model group

#### Analyses of the DEPs

**Fig. 5:**
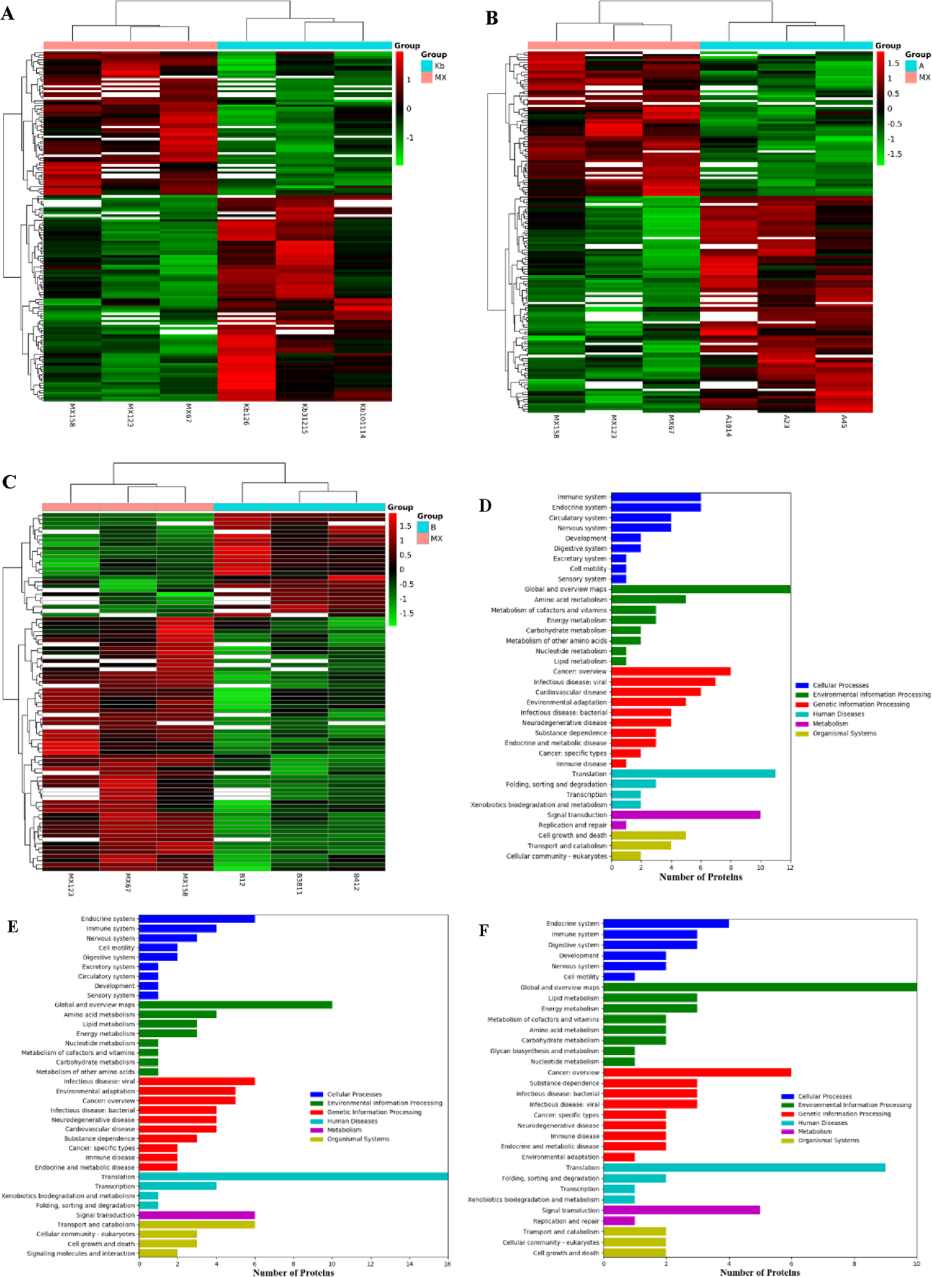
Proteomic analyses of zebrafish larvae samples from the control, model, FA, and FB groups. Heat-map of differential proteins of control vs. model (**a**), model vs. FA (**b**), and model vs. FB (**c**). Rows: differential proteins; columns: zebrafish larvae samples. The rectangle in different colors represented the expression level of proteins: the highest (red), the lowest (green), and the mean (white). The KEGG pathway classification of differential proteins of control vs. model (**d**), model vs. FA (**e**), and model vs. FB (**f**). Rows: number of proteins; columns: KEGG pathway classification

The heat-maps of changes in differential proteins of control vs. model, model vs. FA, and model vs. FB were presented in Fig. 5a–c. The DEPs were categorized according to the following Gene Ontology (GO) classes: biological process, molecular function, and cellular components (Fig. S1). KEGG analysis was further carried out to identify the biological pathways associated with CuSO_4_-induced inflammation in zebrafish, so as to clarify the therapeutic mechanism of FA and FB. As shown in Fig. 5d–f, the identified proteins were involved in a variety of pathways, including cellular processes, environmental information processing, genetic information processing, human diseases, metabolism, and organismal systems, covering a wide range of biological pathways in the inflammatory response and neuromasts damage.

The identified DEPs were imported to the STRING database (https://string-db.org/) for protein-protein interaction (PPI) network constructions (Fig. S2). Compared with the control group, 146 DEPs were identified in the model group, among these DEPs, 101 proteins were found to be involved in the network, 40 proteins were related to each other, and 5 proteins did not display any linkage at a confidence level of string score = 0.4. This network reflected complex functional relationships among the identified proteins in the present study.

### Interactive network construction

**Fig. 6:**
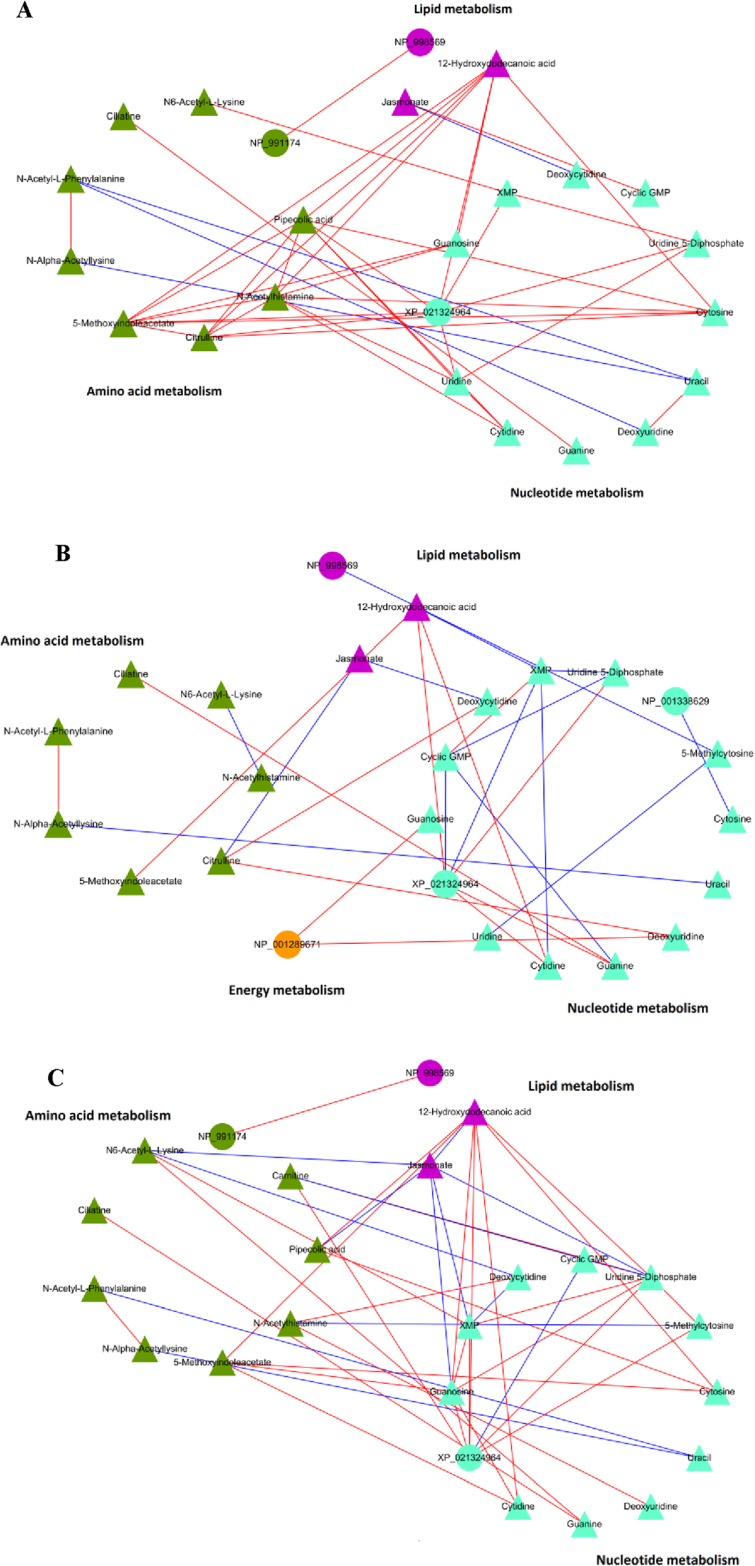
An integrated analysis of metabolomics and proteomics. The interactive network of metabolites and proteins from control vs. model (**a**), model vs. FA (**b**), and model vs. FB (**c**). Triangles and circles in different colors represented the associated metabolites and proteins, respectively

The differentially expressed metabolites and proteins from control vs. model, model vs. FA, and model vs. FB were imported into Cytoscape software to conduct the significant network analyses (Fig. S3). Further integrated analyses of metabolomic and proteomic studies exhibited the altered pathways in response to CuSO_4_-induced acute inflammation, including lipid metabolism, amino acid metabolism, and Nucleotide metabolism (Fig. 6a–c). FA and FB were likely to reverse expressions of metabolites and proteins involved in these metabolic pathways, thus alleviating the injury of hair cells in zebrafish.

### RT-qPCR

**Fig. 7:**
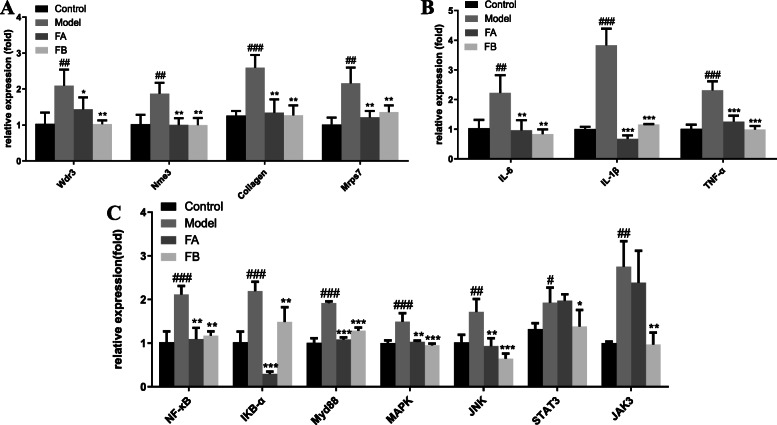
The relative mRNA expression levels in the control, model, FA, and FB groups. **a** The mRNA expressions of Wdr3, Nme3, Collagen, and Mrps7. **b** The mRNA expressions of IL-6, IL-1β, and TNF-α. **c** The mRNA expressions of genes involved in NF-κB, MAPK, and JAK-STAT signaling pathways. ^#^*p* < 0.05, ^##^*p* < 0.01, ^###^*p* < 0.001, compared with the control group; ^*^p < 0.05, ^**^p < 0.01, ^***^p < 0.001, compared with the model group. Data were shown as mean ± S.D, *n* = 35

We used RT-qPCR to detect and quantify the mRNA levels of Wdr3, Nme3, collagen, and Mrps7. We also quantified the inflammatory mediators IL-6, IL-1β, and TNF-α that contribute to the early inflammatory phase, and mediate the recruitment of other immune cells to the damaged areas. Finally, we quantified the genes involved in the NF-κB, MAPK, and JAK-STAT signaling pathways. As a result, gene expressions of Wdr3, Nme3, collagen, and Mrps7 in FA and FB groups were decreased, compared with the model group, which was consistent with proteomic results (Fig. 7a). Moreover, compared with the model group, FA and FB significantly downregulated the expression of IL-6, IL-1β, and TNF-α (Fig. 7B). FB could reverse mRNA expressions of genes involved in NF-κB, MAPK, and JAK/STAT signaling pathways. However, FA mainly exerted an anti-inflammatory effect through NF-κB and MAPK signaling pathways (Fig. 7c).

## Discussion

The inflammatory response is a common and highly regulated biological process in the body that helps to clear harmful irritants and effectively initiate reparation [31]. In the process of inflammation, excessive recruitment and uncontrolled migration of neutrophils to injured sites could result in tissue damage and disease [24]. In this study, we used CuSO_4_ to induce inflammation in zebrafish so that we could monitor the behavior of the neutrophils during the critical transitions phase. CuSO_4_ is a metal chemical that induces neuromasts damage in the zebrafish lateral line system, and can easily induce the characteristics of oxidative stress and inflammation. In addition to their regenerative capacity, the structure, function, and molecular activity in the zebrafish lateral line hair cells are similar to hair cells in mammals [32]. In the present study, neutrophils have significantly accumulated in the damaged neuromasts following exposure to CuSO_4_. However, the number of neutrophils in the neuromasts was clearly reduced when zebrafish were treated with FA or FB, which indicates that by exhibiting anti-inflammatory activities, FA and FB might exert excellent neuroprotective properties against CuSO_4_-induced neuroinflammation.

Excessive ROS and NO production in the body might lead to cell and tissue damage. Therefore, inhibiting this excessive production can effectively impede the progress of the inflammatory response. Such inhibition could be considered as a potential anti-inflammatory drug development target. Exposure to CuSO_4_ significantly increases ROS and NO production in zebrafish. As previously reported, the oxidative stress caused by the overproduction of ROS can continuously stimulate the neurons, leading to their damage and dysfunction [33, 34]. However, FA and FB have markedly inhibited ROS and NO accumulation in the CuSO_4_-induced damaged neuromasts, which further suggests the remarkable neuroprotective properties of FA and FB, preventing the inflammation induced by CuSO_4_ exposure.

Metabolomic analysis has identified multiple metabolites that differed between the control and model groups. Among them, treatment with FA and FB reversed the abnormal expressions of uridine 5-diphosphate, carnitine, Val-Abu-OH, ciliatine, cytidine, benzaldehyde, isobutyryl carnitine, N-acetyl-L-phenylalanine, gamma-glutamyl-leucine, dihydrojasmonic acid, cyclic GMP, 12-hydroxydodecanoic acid, and N-arachidonoyl dihydroxypropylamine. Some metabolites have been reported to participate in the modulation of particular inflammation-related molecules or mediators and play a crucial role in the inflammatory cascade reaction. For example, uridine 5-diphosphate can induce the expression of chemokines such as monocyte chemotactic protein-1 (MCP-1) and macrophage inflammatory protein-1α (MIP-1α) in microglia and astrocytes. These chemokines are the primary effector molecules that mediate the recruitment of inflammatory cells to the damaged tissues [25, 35]. Therefore, the potential role of uridine 5-diphosphate in the inflammatory response is likely to be related to the involvement of chemokines in inflammation. The depletion of carnitine was previously reported to be prevalent in hemodialysis patients, which was assumed to be associated with their inflammatory response in patients [36]. Moreover, exogenous carnitine was required to satisfy energy consumption, which might reflect on carnitine’s indirect modulation of the inflammatory response by regulating energy metabolism. The specific role of energy metabolism in inflammation will be more thoroughly discussed later. Cytidine can be metabolized to uridine through the action of cytidine deaminase. This action activates and increases nucleoside-sensing of the Toll-like receptor 8, which is supported by the overexpression of TNF-α in monocytes or myeloid cells [37, 38]. Thus, the involvement of cytidine in inflammation might be related to the inflammatory cascade reaction induced by the activation of Toll-like receptors.

Further metabolomic pathway enrichment analysis pointed mainly to the involvement of nicotinate and nicotinamide metabolism, energy metabolism, pyrimidine metabolism, and purine metabolism. Most of these pathways are directly or partly involved in inflammation [39–43]. The involvement of nicotinate and nicotinamide metabolism in the inflammatory response might be related to some metabolic enzymes as well as secondary metabolites produced during the metabolic processes. For example, nicotinate phosphoribosyltransferase is an important inflammatory mediator that can bind to Toll-like receptor 4, which in turn induces the activation of inflammasomes and NF-κB. This activation leads to the secretion of the inflammatory mediators IL-1β, IL-8, and TNF-α [40]. Moreover, the identified metabolite nicotinamide ribotide participates in nicotinate and nicotinamide metabolism. This metabolite, also known as nicotinamide mononucleotide (NMN), can be converted into the principal precursor of nicotinamide adenine dinucleotide (NAD^+^) through transamination [44, 45]. To activate Sirtuin 1 (*SIRT1*), NAD^+^ regulates the ratio between NAD^+^ and nicotinamide adenine dinucleotide (NADH). *SIRT1*, in turn, positively regulates NF-κB expression, and NF-κB is a crucial transcription factor involved in pro-inflammatory cytokines generation [46, 47]. Presently, it can be speculated that FB might decrease NAD^+^ synthesis by inhibiting the expression of nicotinamide ribotide. This cascade may further result in an inactivation of *SIRT1* and the NF-кB signaling pathway, along with a decrease in the release of inflammatory factors.

Differentially expressed metabolites such as cytidine, cytosine, deoxyuridine, uracil, XMP, dIMP, cyclic GMP, and guanosine were identified in the present study. They are all involved in pyrimidine and purine metabolism. To reach a reasonable comprehension of the involvement of pyrimidine and purine metabolism in the inflammatory response, the participation of pyrimidine and purine nucleotide receptors (P2Y receptors) had to be well explained [43]. That is, P2Y receptors are present practically in all immune cells, and can specifically mediate the inflammatory response to various cell injuries by recognizing pyrimidine and purine nucleotides [43]. For example, the P2Y6 receptor shows the highest expression in macrophages, dendritic cells, and microglia. The activation of this receptor could lead to immune phenotypes such as macrophage and microglial dysfunction, and inactivation of effector T cells. It also plays a significant role in vascular inflammation [48–50]. Moreover, the inhibition of P2Y6 receptor was shown to be accompanied by a decrease in the release of IL-8, IL-1α, and IL-6, a release that was induced by monosodium urate crystals in human keratinocytes [51]. Therefore, the significant role of pyrimidine and purine metabolism in the inflammatory response might be closely related to the immune functions played by various P2Y receptors. Consequently, it can be speculated that FA and FB might exert their anti-inflammatory effects by regulating these metabolites, and through them modulating cellular immune functions and the release of various inflammatory mediators.

Energy consumption also plays an essential role in the development of inflammation. In the present study, Starch and sucrose metabolism, amino sugar and nucleotide sugar metabolism, galactose metabolism, as well as the pentose phosphate pathway (PPP) and the glycolysis, have all been shown to be related to energy metabolism. During the inflammatory response, there is an increased demand for energy by the immune cells. This extra energy is needed to satisfy the demand by housekeeping functions and multiple immune cells-specific functions. Such functions include cell migration, cytokinesis, antigen processing and presentation, and lymphocyte activation [38, 52]. The inflammatory response can increase energy consumption by as much as 25–60% [53, 54]. As reported, the use of drugs to regulate the energy metabolism of immune cells could be a potential treatment strategy [55, 56]. For example, glucose-6-phosphate (G6P) is at the intersection of multiple energy metabolism pathways, through which it can be converted into PPP for nicotinamide adenine dinucleotide phosphate (NADPH) and ribose-5-phosphate. The activation of PPP contributes to the inhibition of P53. This inhibition leads to increased glucose consumption, NADPH production, and biosynthesis [57]. In our study, G6P was found to be significantly upregulated in the model group relative to the control group. Treatment with FB remarkably downregulated G6P, suggesting that FB might serve as an anti-inflammatory agent by regulating the expression of G6P, as well as the reduction in energy consumption. Meanwhile, the relieved damage in the FB group might also contribute to the decreased production of G6P and the reduced requirement of energy consumption. That is, in CuSO_4_-induced acute inflammation, zebrafish need more energy to conduct a series of inflammation-related activities and immune cells activation. They also need it to repair the damaged tissues. Zebrafish larvae in the FA and FB groups tended to require less energy, which was basically used to maintain housekeeping functions. In other words, the reduced energy consumption might be secondary to the reduced inflammatory response in the FA and FB groups.

The proteomic analysis has identified 146 DEPs when comparing the model group and control groups. Among these DEPs, 51 and 18 proteins were reversed in the FA and FB groups, respectively. The KEGG pathway classification showed that the DEPs covered a variety of cellular processes and metabolic pathways involved in the inflammatory response and neuromast damage. The identified DEPs participate in multiple biological processes and categories such as immune system and infectious disease, nervous system and neurodegenerative disease, signal transduction, and cell growth and death. Our data suggest that the exposure to CuSO_4_ could have damaged both the immune and the nervous systems of the zebrafish. Such damage involves a variety of signal transduction as well as cell death and regeneration. The identified proteins also participate in various metabolomic pathways, including amino acid metabolism, lipid metabolism, nucleotide metabolism, and energy metabolism. These pathways could influence multiple inflammation-related processes, as will be further elaborated in the discussion below on integrating the metabolomic and proteomic analyses. The changes in expression of the identified DEPs that are actively or passively involved in the inflammatory response, including collagen (col2a1b, col9a2, col9a1b), Wdr3, Mrps7, and Nme3, have all been reversed both in the FA and FB groups [58–61]. Previous studies have shown that the organization of ROS, NF-κB, and collagen at the injury margins is involved in promoting wound healing [62]. High levels of TNF-α might lead to an excessive inflammatory response, along with increased deposition of collagen in fibroblast-treated wounds [63]. In addition to the generation of collagen, remodeling its structure, as induced by tissue damage, also plays a crucial role in the repair process. This remodeling might be achieved by proteases such as the matrix metalloproteinase (MMP) [64, 65]. The critical role of MMP9 in collagen reorganization and regeneration has been previously reported. One report suggested that MMP9 could aggravate wound damage in chronic injury, but promote wound healing and regeneration in acute injury [66]. This implies a critical role for collagen in tissue damage and repair. Our findings suggested that both FA and FB might guide treatments focused on prevention (neuroprotection) or repair (anti-inflammatory) of CuSO_4_-induced hair cells damage in the zebrafish larvae by modulating the productions of collagen.

Mitochondrial ribosomal protein (MRP) subunits are nuclear-encoded and were crucial for mitochondrial functions and mitochondrial-encoded protein synthesis. These subunits were also reported to be involved in mitochondrial diseases [61, 67]. Mrps7, a 12S ribosomal RNA binding subunit, is necessary for the assembly of small ribosomal subunits. Studies have confirmed that Mrps7 mutations could cause mitochondrial respiratory chain dysfunction and congenital sensorineural deafness [68]. Mitochondria are an essential component of the innate inflammatory response. Their dysfunction was shown to be a vital trigger of inflammation and could result in metabolic alteration and mitochondrial damage-induced release of inflammatory mediators [69]. Thus, we speculated that alterations in Mrps7 expression induced by CuSO_4_ exposure might be related to mitochondrial dysfunction in the zebrafish. This dysfunction could be the main source of ROS following the increase in energy demands and efficient mitochondrial function-dependent excessive inflammatory activities [70].

As a member of the WD repeat-containing proteins, Wdr3 participates in several cellular processes such as cell cycle progression and signal transduction [71, 72]. Studies have confirmed that some Wdr proteins participate in the MAPK and STAT3 signaling pathways [73]. Moreover, the expression of misfolded Wdr proteins in dendritic cells could activate the inflammasome and result in a substantial release of IL-18 [60]. Here, we can speculate that FA and FB may have reversed the abnormal expression of Wdr3, which in turn regulate Wdr proteins-mediated cell cycle progression, signal transduction, and the release of inflammatory mediators from the inflammasomes. Nme3, also known as NM-23, participates in a variety of physiological and pathological cellular processes such as differentiation, development, cellular signaling, and cellular function. It was reported that Nm23-H1 could interact with p53 and positively mediate the apoptosis and cell cycle arrest that was induced by p53 [74]. Besides, Nme3 was confirmed to modulate pro-inflammatory transcription by activating NF-κB [75]. This modulation activates the Toll-like receptor 5-mediated NF-κB signaling pathway in a MyD88-dependent manner [59]. In the present study, both FA and FB downregulated Nme3 expression, suggesting that FA and FB might possess a regulatory effect on cellular signaling and function.

Integration of the metabolomic and proteomic analyses indicates that the lipid metabolism, amino acid metabolism, and nucleotide metabolism are significantly altered as a result of CuSO_4_ exposure. The analyses helped to form a more comprehensive understanding of the metabolic pathways involved in CuSO_4_-induced inflammation in zebrafish. Moreover, pathways identified through the metabolomic analysis, including the pyrimidine and purine metabolism, are indispensable during the metabolism of lipids, amino acid, and nucleotides. Through these metabolic pathways, they perform their crucial role in lipids activation, energy conservation, and nucleotide synthesis [76]. Presently, lipids are considered as an effective signaling molecule that regulates multiple cellular responses. A variety of lipid-derived mediators are involved in the regulation of inflammation and the response to infection [77, 78]. For example, the lipid-derived mediators, prostaglandin E2 (PGE_2_) and prostacyclin (PGI_2_), function as vasodilatation agents during the initial stage of inflammation. These mediators help to recruit various immune cells from the blood, including neutrophils and macrophages, and mobilize them to the infected or damaged tissues [79]. Therefore, it can be speculated that FA and FB might exert therapeutic effects by regulating lipid metabolism. In turn, this regulation might affect the role of lipid-derived mediators in the inflammatory response. Amino acid metabolism is also considered to mediate the immune response in mammals. Their metabolism might be necessary for pivotal enzymes, and their degradation could be crucial immune checkpoints in autoimmunity [80]. The activation of enzymes such as indoleamine 2,3-dioxygenase 1 (IDO1) and arginase 1 (ARG1) could induce an immunoregulatory effect on dendritic cells and inflammatory cells. This effect could coordinate the immune response and resistance to inflammation [81, 82]. Additionally, nucleotide metabolism could occur under inflammatory conditions and following tissue injury [83]. The various nucleotides released to the extracellular domain, including ATP, ADP, and UDP, could specifically bind with individual P2Y receptors. These receptors, in turn, participate in handling tissue damage and the recruitment of immune cells [83–85]. Therefore, FA and FB might exert their anti-inflammatory activities by modulating the amino acid metabolism and nucleotide metabolism, and thus reducing the immune response and alleviating the damage caused to the neuromasts.

Moreover, in the present study, we also detected changes in mRNA expression of genes involved in the NF-κB, MAPK, and JAK-STAT inflammatory signaling pathways, and in the production or release of inflammatory cytokines such as IL-6, IL-1β, and TNF-α. NF-κB, MAPK, and JAK-STAT pathways are involved in the signal transduction of the inflammatory response, including regulation of the secretion of various chemokines and inflammatory cytokines. These contribute to an amplified inflammatory response and worsening of the tissue damage [86–88]. Our findings suggested that FA and FB might ameliorate inflammation by negatively interfering with these signaling pathways. The inflammatory cytokines IL-6, IL-1β, and TNF-α are the most studied pro-inflammatory cytokines. They could contribute to the early inflammatory phase and participate in multiple inflammation-related processes. These effects are partly mediated by their crucial role in the recruitment of additional inflammatory cells to the infected or injured tissues [3]. Furthermore, IL-6, TNF-α, and IL-1β were also reported to play a significant role in neurocyte damage-induced neuropathic pain [89]. Immediately after nerve injury, neutrophils and macrophages are recruited to the injured area, resulting in a substantial release of cytokines such as IL-6, TNF-α, and IL-1β. These cytokines can then directly regulate the activity of the neurons [89, 90]. In the present study, CuSO_4_ was used to specifically induce neuromasts damage in the lateral line system of zebrafish. This damage initiated the infiltration of immune cells such as neutrophils, and possibly also the release of inflammatory mediators such as IL-6, TNF-α, and IL-1β. However, both FA and FB were found to inhibit the recruitment of neutrophils and the secretion of inflammatory mediators induced by CuSO_4_. Thus, FA and FB were able to reduce inflammation and alleviate neuromasts damage. It was further shown that through anti-inflammatory activities, FA and FB exhibit neuroprotection against the CuSO_4_-induced neuroinflammatory response.

Presently, we applied the integration of metabolomics and proteomics to explore the anti-inflammatory properties and mechanisms of FA and FB against CuSO_4_-induced neuromasts damage in zebrafish larvae. Our results indicated that both FA and FB significantly inhibited the injury of CuSO_4_-induced hair cells in zebrafish neuromasts. Based on our study, we presumed that FA and FB might exhibit neuroprotective properties in the damage of CuSO_4_-induced zebrafish neuromasts, evidenced by the reduced recruitment of neutrophils and inhibited expressions of ROS, NO, and inflammatory mediators including IL-6, IL-1β, and TNF-α. Metabolomic and proteomic analyses indicated that FA and FB might share similar anti-inflammatory mechanisms to some extent because of the same maternal nucleus structure. The different substituents of hydroxyl groups of FA and FB might indicate a minor difference in anti-inflammatory activities: FA exhibited no effect on JAK/STAT signaling pathway. On the contrary, FB could reverse the overexpression of STAT3 and JAK3.

**Fig. 8:**
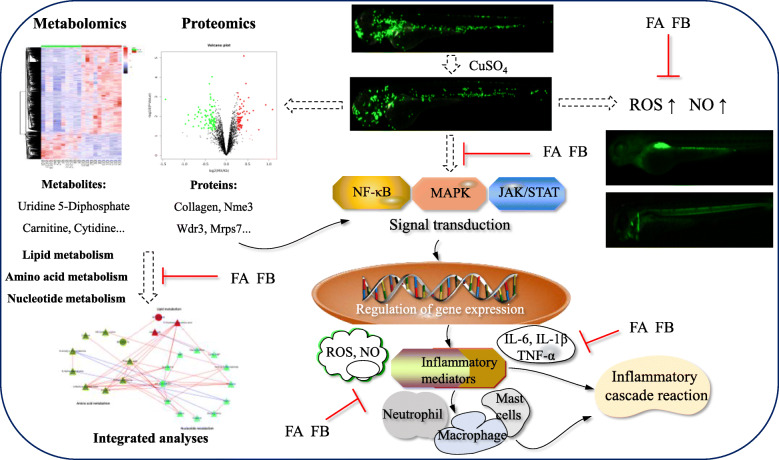
The possible mechanism of CuSO_4_-induced inflammation in zebrafish and the action diagram of FA and FB. FA and FB administration resulted in reductions of ROS and NO, alterations of various metabolites, proteins, and gene products induced by CuSO_4_, thereby reducing inflammation. This diagram showed the multi-component and multi-regulatory therapeutic mechanism of FA and FB against CuSO_4_-induced inflammation in zebrafish

In summary, we proposed a potential mechanism of CuSO_4_-induced inflammation and the action diagram of FA and FB (Fig. 8). FA and FB administration resulted in reductions of ROS and NO, gene expressions of Wdr3, collagen, Nme3 and Mrps7, and other inflammatory mediators induced by CuSO_4_, thus reducing inflammation. As discussed above, a series of metabolites, metabolic pathways, proteins, and gene products were altered in response to CuSO_4_ exposure. However, FA and FB treatments significantly reversed these changes through a complicated regulatory mechanism. Our findings suggested that these identified metabolites and proteins may serve as potential anti-inflammatory biomarkers. FA and FB exhibited excellent anti-inflammatory activities against CuSO_4_-induced inflammation in zebrafish. Due to the multi-component and multi-regulatory process in response to CuSO_4_ exposure, FA and FB might influence downstream conduction process by affecting some of the upstream inflammatory processes. For instance, reductions in NO and ROS may lead to secondary effects on their downstream products, such as the decreased expressions of downstream inflammatory mediators IL-6, IL-1β, and TNF-α via modulating specific inflammatory signaling pathways. Accordingly, the reversed expressions of Collagen, Nme3, Wdr3, and Mrps7 in FA and FB group against CuSO_4_ exposure may also be secondary to the regulation of some metabolic pathways upstream. Also, the fact that zebrafish were both pre- and post-treated with FA or FB may provide another potential therapeutic mechanism, that is, pretreatment may attenuate the initiation of inflammation wherein post-treatment may inhibit the inflammatory cascade as well. Both administration methods will inhibit the inflammatory response through different mechanisms and have different therapeutic significance, which can be further studied in the future.

## Conclusion

In conclusion, we analyzed the inhibitory effect and therapeutic mechanism of FA and FB against CuSO_4_-induced inflammation in zebrafish using UPLC-MS/MS based metabolomics and TMT-6 Plex based proteomics for the first time. The alterations of identified metabolic intermediates and protein biomarkers might help to elucidate the anti-inflammatory mechanisms of FA and FB against CuSO_4_-induced inflammation in zebrafish. Our results also provided a potential novel and sequential mechanism for the initiation of inflammation induced by CuSO_4_ and suggested a multi-component and multi-regulatory therapeutic mechanism for FA and FB.

## Supplementary information

**Additional file 1: Table S1.** Primers used for RT-qPCR.

**Additional file 2: Table S2.** Metabolic pathways associated to varied influences of FA and FB in CuSO_4_-induced zebrafish inflammation.

**Additional file 3: Figure S1.** GO analysis of differentially-expressed proteins.

**Additional file 4: Figure S2.** Protein-protein interactions of differentially expressed proteins.

**Additional file 5: Figure S3.** Interactive network construction by integrated metabolomics and proteomics analysis.

## Data Availability

The datasets generated and/or analyzed in the current study are available from the corresponding author on reasonable request.

## Abbreviations

FA: Forsythiaside A
FB: Forsythiaside B
IL-6: Interleukin-6
TNF-α: Tumor necrosis factor-α
IL-1β: Interleukin-1β
dpf: Days postfertilization
DCF-DA: 2,7-dichlorodihydrofluorescein diacetate
DAF-FMDA: Diamino fluorophore 4-amino-5-methylamino-2′,7′-difluorofluorescein diacetate
ROS: Reactive oxygen species
NO: Nitric oxide
LC-MS: Liquid chromatography-mass spectrometry
HPLC: High-performance liquid chromatography
ESI-MSn: Electrospray ionization mass detection
m/z: Mass to charge ratio
MS/MS: Mass spectrometry/mass spectrometry
HCD: Higher energy collisional dissociation
UPLC-MS/MS: Ultra-performance liquid chromatography-mass spectrometry/mass spectrometry
OPLS-DA: Orthogonal projections to latent structures discriminant analysis
VIP: Variable importance in the projection
ppm: Part per million
HMDB: Human metabolome database
KEGG: Kyoto Encyclopedia of Genes and Genomes Database
TMT: Tandem mass tags
RP: Reverse phase
NF-κB: Nuclear transcription factor-kappa B
MAPK: Mitogen-activated protein kinases
JAK-STAT: Janus kinase/signal transducer and activator of transcription
OD: Optical density
BPC: Base peak chromatogram
PCA: Principal component analysis
PLS-DA: Partial least squares-discriminant analysis
DEPs: Differentially expressed proteins
FDR: False discovery rate
RT-qPCR: Reverse transcription quantitative real-time PCR
GO: Gene Ontology
PPI: Protein-protein interaction
MCP-1: Monocyte chemotactic protein-1
MIP-1α: Macrophage inflammatory protein-1α
NMN: Nicotinamide single nucleotide
NAD+: Nicotinamide adenine dinucleotide
NADH: Nicotinamide adenine dinucleotide
SIRT1: Sirtuin 1
P2Y: Pyrimidine and purine nucleotide
PPP: Pentose phosphate pathway
G6P: Glucose-6-phosphate
NADPH: Nicotinamide adenine dinucleotide phosphate
MMP: Matrix metalloproteinase
MRP: Mitochondrial ribosomal protein
IDO1: Indoleamine 2,3-dioxygenase 1
ARG1: Arginase 1
PGE_2_: Prostaglandin E2
PGI_2_: Prostacyclin
