# Supplementary material for: Exploration of anti-inflammatory mechanism of forsythiaside A and forsythiaside B in CuSO4-induced inflammation in zebrafish by metabolomic and proteomic analyses
Source: J Neuroinflammation. 2020 Jun 3;17:173. doi: 10.1186/s12974-020-01855-9 (PMC7271515; doi:10.1186/s12974-020-01855-9)
Supplement: Supplementary file 1 — Additional file 1: Table S1. Primers used for RT-qPCR. [file 12974_2020_1855_MOESM1_ESM.docx]

**Table S1** Primers used for quantitative real-time PCR

| **Gene** | **Forward primer** | **Reverse primer** |
| --- | --- | --- |
| WDR3 | TGAAAGCTGCCGAGCGAATA | TAACTTTTTCCCCGCTGCCT |
| MRPS7 | GCCTCCGTCAGGCATTTACT | CTGGGGCATCTTTTCTGGGT |
| NME3 | AGCACTGGATCTACGCTTGA | GCTCTGTGTGTGTGTTTGATGATG |
| Collagen  STAT3 | CCATGCTGTACAGTGTCGTGA | AGTGGGACTGGCCTTCTTTC |
|  | ATCGACCTTGAGACGCACTC | CCCATGCGTTTGGCATTTGA |
| JAK3 | AACAGAGCGAGCAGCAGAGAG | GTGTGACCACCCTTCCTTCC |
| NF-κB | GAGCCCTTTGTGCAAGAGAC | TGGGATACGTCCTCCTGTTC |
| MyD88 | GAGGATGGTGGTGGTCATCT | CGACAGGGATTAGCCGTTTA |
| IκBα | TTTCGGAGGAGATGGAGAGA | CTGTTCAGGTACGGGTCGTT |
| MAPK | TTACCTGCTGTCCCTTCC | TCCTCCACCTCAATCCTC |
| JNK | GGGAATAGTGTGTGCTGGATATGATG | TGGTTCTGGAAGGGTCTGCTGAG |
| IL-1β | TGGACTTCGCAGCACAAAATG | GTTCACTTCACGCTCTTGGATG |
| IL-6 | ATGACGGCATTTGAAGGG | GCAGCGGTCTGAAGGTTT |
| TNF-α | GCTTATGAGCCATGCAGTGA | TGCCCAGTCTGTCTCCTTCT |
| GAPDH | GGATCTGACAGTCCGTCTTGAGAA | CCATTGAAGTCAGTGGACACAACC |
