# Supplementary material for: Exploration of anti-inflammatory mechanism of forsythiaside A and forsythiaside B in CuSO4-induced inflammation in zebrafish by metabolomic and proteomic analyses
Source: J Neuroinflammation. 2020 Jun 3;17:173. doi: 10.1186/s12974-020-01855-9 (PMC7271515; doi:10.1186/s12974-020-01855-9)
Supplement: Supplementary file 2 — Additional file 2: Table S2. Metabolic pathways associated to varied influences of FA and FB in CuSO4-induced zebrafish inflammation. [file 12974_2020_1855_MOESM2_ESM.docx]

**Table S2** Metabolic pathways associated to varied influences of FA and FB in CuSO_4_-induced inflammation in zebrafish

| **Metabolic pathways** | **Groups** | **Total** | **Hits** | **LOG(p)** | **Impact** |
| --- | --- | --- | --- | --- | --- |
| Nicotinate and nicotinamide metabolism | Model vs FB | 14 | 1 | 1.6565 | 0.16043 |
| Starch and sucrose metabolism | Model vs FB | 22 | 1 | 1.2597 | 0.15951 |
| Amino sugar and nucleotide sugar metabolism | Model vs FB | 37 | 1 | 0.84082 | 0.08988 |
| Pyrimidine metabolism | Model vs FA | 41 | 1 | 1.769 | 0.00737 |
|  | Model vs FB | 41 | 3 | 3.8559 | 0.0706 |
| Pentose phosphate pathway | Model vs FB | 19 | 1 | 1.3857 | 0.06982 |
| Purine metabolism | Model vs FB | 66 | 4 | 4.2433 | 0.05238 |
| Glycolysis or Gluconeogenesis | Model vs FB | 26 | 1 | 1.1199 | 0.0286 |
| Galactose metabolism | Model vs FB | 26 | 1 | 1.1199 | 0.02406 |
