# Supplementary material for: Exploration of anti-inflammatory mechanism of forsythiaside A and forsythiaside B in CuSO4-induced inflammation in zebrafish by metabolomic and proteomic analyses
Source: J Neuroinflammation. 2020 Jun 3;17:173. doi: 10.1186/s12974-020-01855-9 (PMC7271515; doi:10.1186/s12974-020-01855-9)
Supplement: Supplementary file 3 — Additional file 3: Figure S1. GO analysis of differentially-expressed proteins. [file 12974_2020_1855_MOESM3_ESM.docx]

**Fig. S1** GO analysis of differentially expressed proteins of control vs. model **(A)**, model vs. FA **(B)**, model vs. FB **(C)**. Rows: Number of proteins; Columns: GO functional classification.
