# Supplementary material for: Exploration of anti-inflammatory mechanism of forsythiaside A and forsythiaside B in CuSO4-induced inflammation in zebrafish by metabolomic and proteomic analyses
Source: J Neuroinflammation. 2020 Jun 3;17:173. doi: 10.1186/s12974-020-01855-9 (PMC7271515; doi:10.1186/s12974-020-01855-9)
Supplement: Supplementary file 4 — Additional file 4: Figure S2. Protein-protein interactions of differentially expressed proteins. [file 12974_2020_1855_MOESM4_ESM.docx]

**Fig. S2** Protein-protein interactions of diferentially expressed proteins from control vs. model **(A)**, model vs. FA **(B)**, model vs. FB **(C)**. The circle in different color represented the relative expression level of proteins. Red: up-regulation; Green: down-regulation; White: mean.
