# Supplementary material for: Exploration of anti-inflammatory mechanism of forsythiaside A and forsythiaside B in CuSO4-induced inflammation in zebrafish by metabolomic and proteomic analyses
Source: J Neuroinflammation. 2020 Jun 3;17:173. doi: 10.1186/s12974-020-01855-9 (PMC7271515; doi:10.1186/s12974-020-01855-9)
Supplement: Supplementary file 5 — Additional file 5: Figure S3. Interactive network construction by integrated metabolomics and proteomics analysis. [file 12974_2020_1855_MOESM5_ESM.docx]

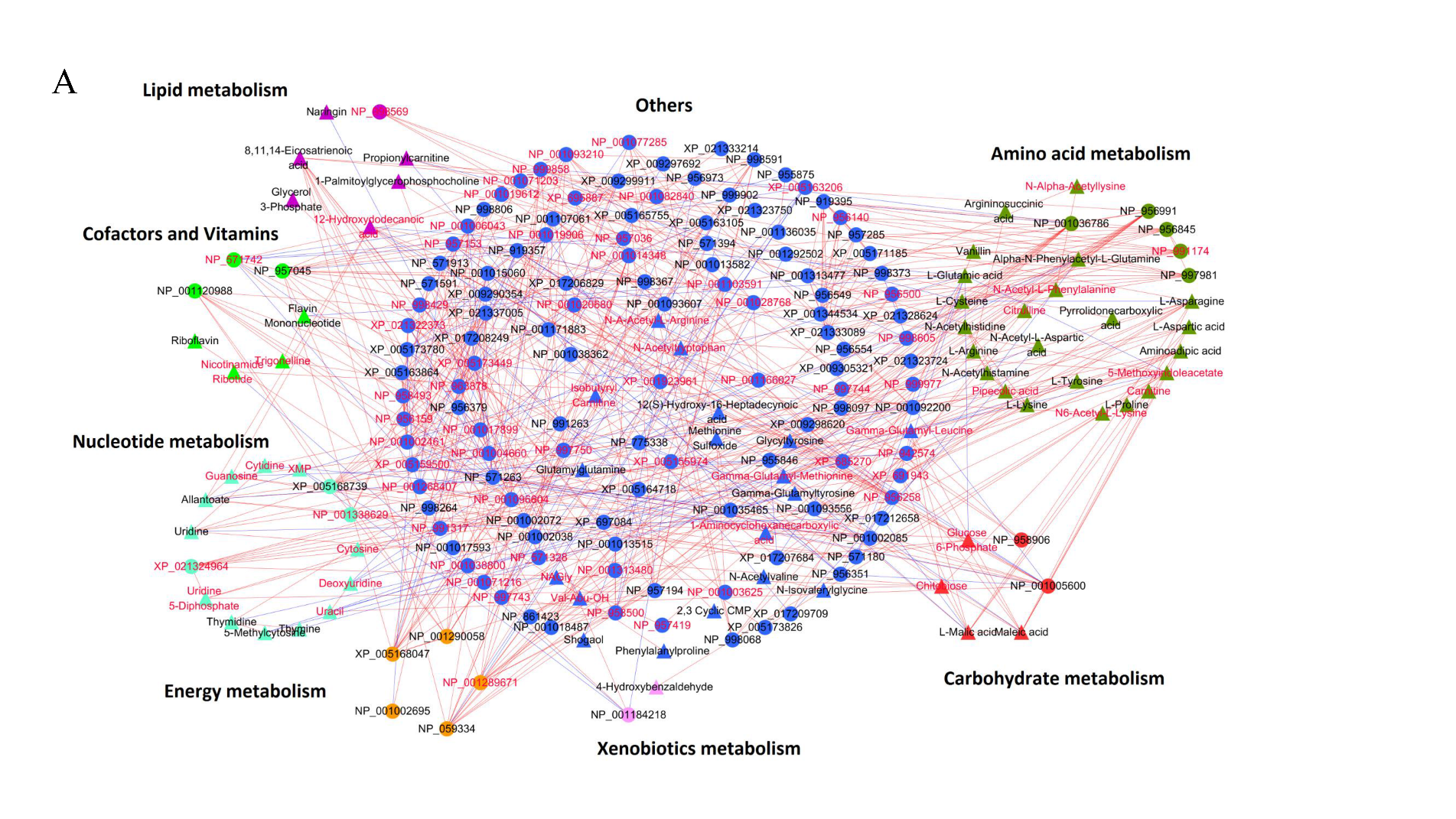

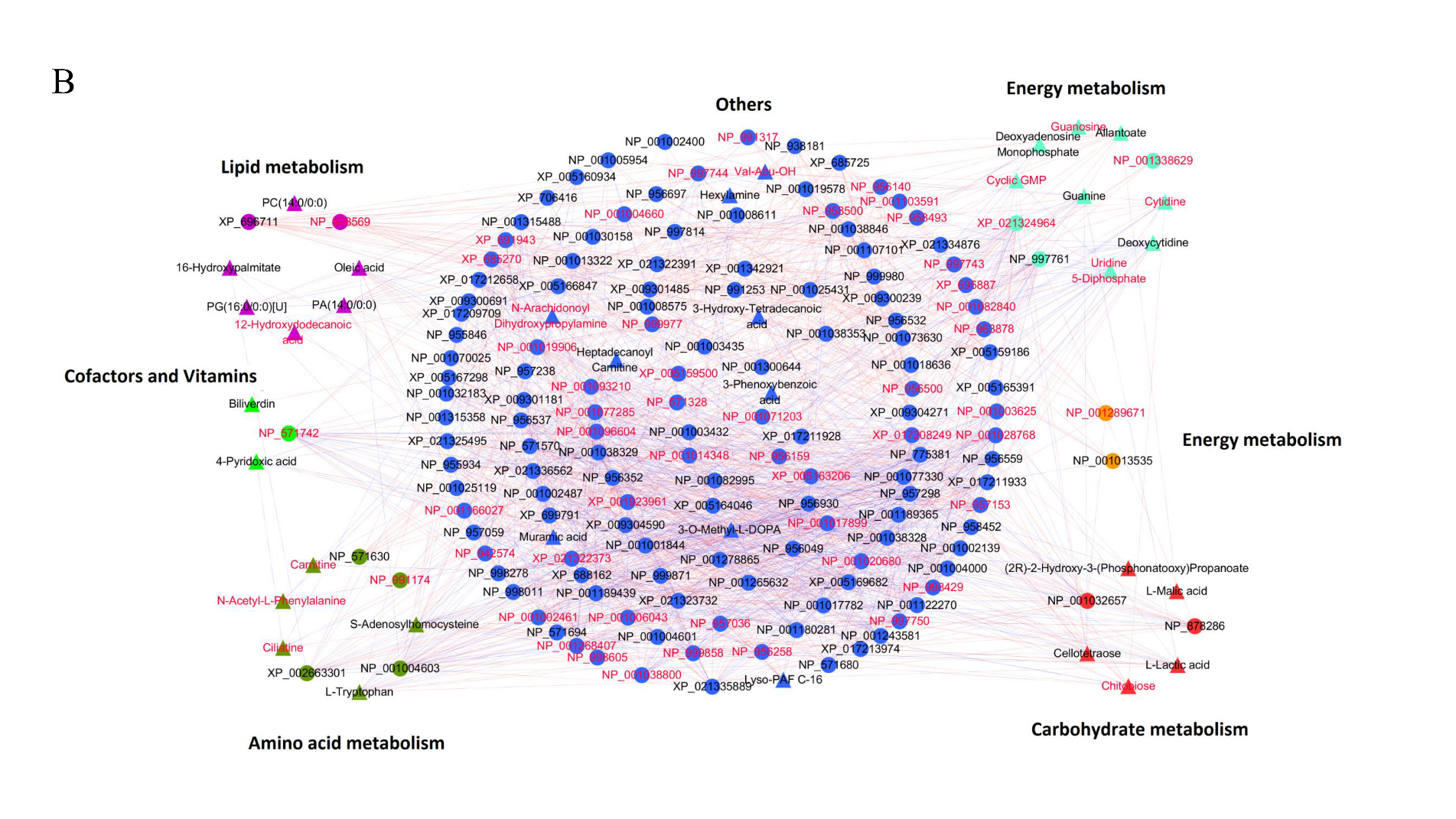

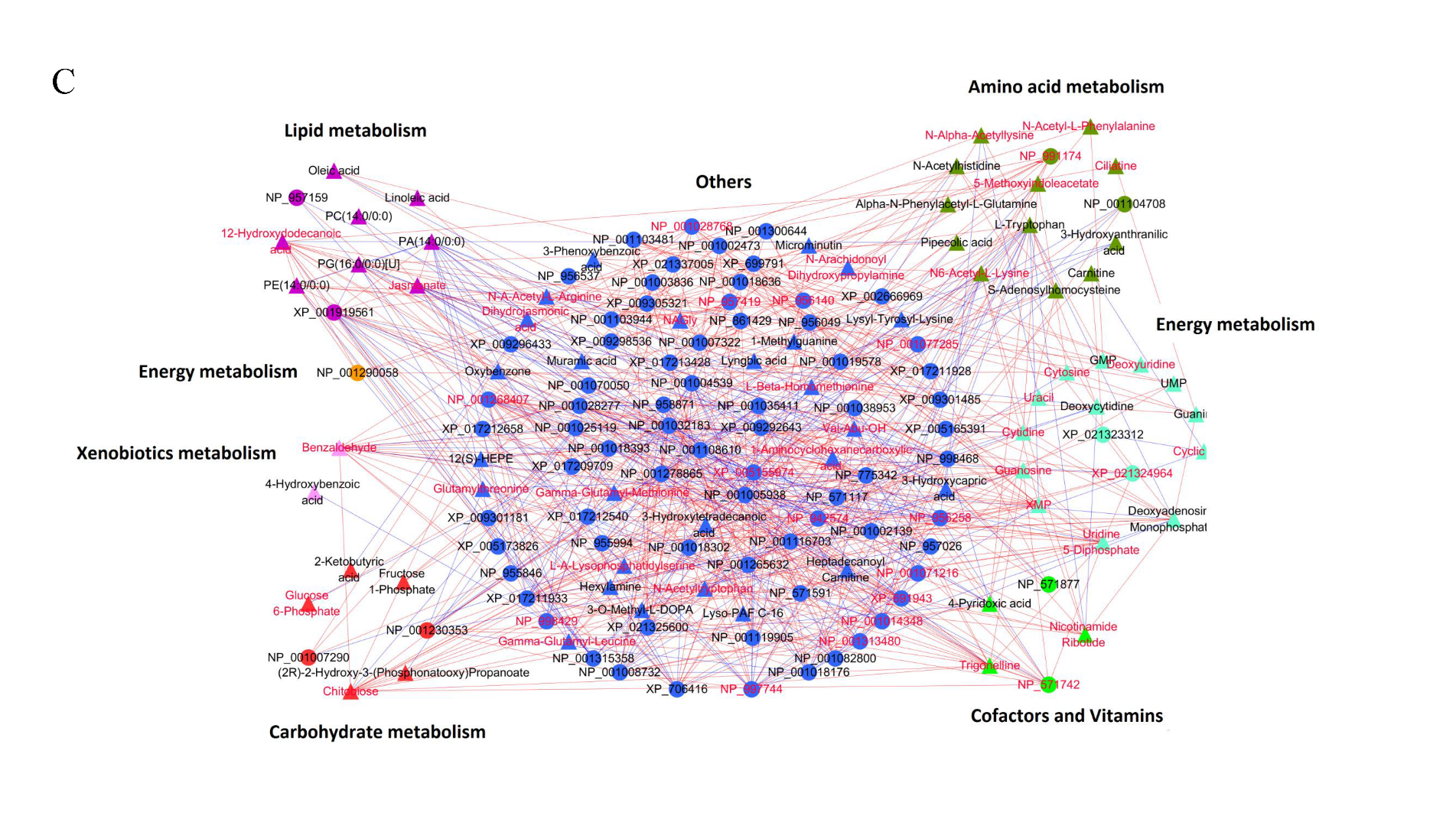


**Fig. S3** Interactive network construction by integrated metabolomics and proteomics analysis. Interactive network of the metabolites and proteins of model compared with control **(A)**, FA **(B)**, and FB **(C)**. Triangles and circles in different colors represented metabolites and proteins in different metabolic pathways. The metabolites and proteins in red represented identified biomarkers in the present study.
